# Effect of guided counseling on dietary practices of pregnant women in West Gojjam Zone, Ethiopia

**DOI:** 10.1371/journal.pone.0233429

**Published:** 2020-05-26

**Authors:** Yeshalem Mulugeta Demilew, Getu Degu Alene, Tefera Belachew

**Affiliations:** 1 School of Public Health, College of Medicine and Health Sciences, Bahir Dar University, Bahir Dar, Ethiopia; 2 Department of Nutrition and Dietetics, Faculty of Public Health, Jimma University, Jimma, Ethiopia; Weill Cornell Medical College in Qatar, QATAR

## Abstract

**Background:**

In Ethiopia, although nutrition education has been given during pregnancy, most women have inadequate nutrient intakes. As a result, the prevalence of malnutrition is high during pregnancy. In this study, we set out to evaluate the effect of guided counseling based on the health belief model and the theory of planned behavior on the dietary practices of pregnant women.

**Methods:**

A two-arm parallel cluster randomized controlled community trial was carried out among pregnant women in West, Gojjam Zone, Ethiopia from May 1, 2018, to April 30, 2019. A total of 346 and 348 pregnant women were recruited from the intervention and control clusters, respectively. Of which endline data were collected from 313 and 332 pregnant women in the intervention and control groups, respectively. Each woman in the intervention group attended four counseling sessions. Women in the control group attended the routine nutrition education given by the health care system. Data were collected using a structured questionnaire. McNemar test and Generalized Estimating Equations were used to evaluate the intervention effect.

**Results:**

The average difference of appropriate dietary practice between the two groups was 30.7%. After controlling for the possible confounders, women in the intervention group had 7.2 times [AOR = 7.187, 95% CI: (4.49, 11.49)] higher odds of having appropriate dietary practices compared with the control group. Dietary diversity and meal frequency of counseled women were 7 [AOR = 6.994, 95% CI: (4.59, 10.66)] and 8 [AOR = 8.146, 95% CI: (5.377, 12.341)] times higher than dietary diversity and meal frequency of women in the control group, respectively.

**Conclusion:**

Counseling based on the health belief model and the theory of planned behavior is an effective approach in increasing the proportion of women who had appropriate dietary practices. Thus, these findings suggest the need for employing trimester based counseling using the HBM and the TPB to improve the dietary practices of pregnant women.

**Trial registration:**

The trial was registered in Clinical Trials.gov (NCT03627156).

## Introduction

Poor maternal diet during pregnancy determines fetal growth, leading to low birth weight, which has negative repercussions on child survival and economic productivity later in life [1]. Despite its serious consequences, in Ethiopia, 13.5% of newborn babies weighed less than 2500g at birth. Moreover, the magnitude of maternal and child mortality is high in the country [2].

These maternal and childhood problems are hypothesized to be due to inadequate nutrient intake during pregnancy [3–6]. As reported by different scholars, diets of Ethiopian pregnant women were inadequate in quantity and poor in quality with low energy and nutrient content [3–5].

The primary determinant for poor nutrient intake is insufficient knowledge of pregnant woman on diet during pregnancy [3, 4, 7]. Since, improving maternal diet is the most appealing and sustainable strategy for promoting maternal and child health [8], the Ethiopian government recommends nutrition counseling during pregnancy [9].

Health professionals and health extension workers give nutrition education to pregnant women during antenatal care visits and in the community. They have been educating pregnant women to take one additional meal from foods available at home compared to their meals before pregnancy. Despite this, the existing nutrition education which is given by the health system isn’t successful in bringing behavior change [10].

This lack of success in the existing nutrition education may be due to poor counseling practices of health professionals and health extension workers. Because, the routine practice is advising pregnant women to eat one additional meal from foods available at home compared to their meals before pregnancy [11, 12].

This type of education is unclear to the women since what is available at home is not specific and varies across households. Moreover, taking one additional meal may not be enough for all pregnant women as nutrient requirements vary for different women and the trimester of pregnancy. Furthermore, dietary habits are also different from woman to woman. Therefore, modifying the method of nutrition education seems to have a paramount benefits to improve maternal nutrition [13].

Guided counseling is a type of behavior change intervention using professional guidance on change of behavior based on models and theories [14]. Health promotions that are supported by behavioral models and theories bring positive results on improving the dietary practices of pregnant women [15–18]. Evidence suggests the need for using multiple theories to change unhealthy behavior as the causes are multifactorial in nature [15, 19]. In this study, pregnant women were counseled based on the health belief model (HBM) and the theory of planned behavior (TPB) [13, 20].

The HBM is an interpersonal model of health applied to encourage positive behavior [21]. It explains the reason why some people take measures to promote health, while others don’t. Its structure consists of perceived susceptibility, severity, benefits, and barriers to a specific behavior. Cues to action and self-efficacy are also components of the HBM [13, 16, 20, 21].

The first element of the TPB is an intention that directly affects a given behavior. Other elements of this theory are attitude, subjective norms, and behavioral control. These constructs affect the intention of women to words having a healthy diet [22, 23]. Hence, this intervention was designed to evaluate the effect of guided counseling based on the HBM and the TPB on improving the dietary practices of pregnant women.

## Methods

### Study setting, design, and participants

This study was carried out in West Gojjam Zone, Amhara Region, Ethiopia. The number of estimated pregnant women was 61,072. Pregnant women before 16 weeks of gestation who had planned to stay in the study area until delivery were enrolled in this trial. Women with hypertension or diabetes mellitus were excluded from the study. The full description of the study area and participants is described elsewhere [12].

A two-arm parallel cluster randomized controlled community trial was conducted from May 1, 2018, to April 30, 2019. The study was carried out in accordance with the Declaration of Helsinki and the requirement of good clinical practice [24].

The sample size was determined by the G power 3.1.9.2 program with a power level of 85% for Fisher’s exact test with a 5% alpha. The expected proportion of pregnant women with appropriate dietary practice (P1) was 0.393 [4]; while P2 was 0.547 [10, 25], assuming a difference 15.4% between p1 and P2. Since cluster randomization was used, the calculated sample size was multiplied by 2 to adjust the design effect and a 10% loss to follow up was added. The final sample sizes were **356** in the intervention and control groups each.

Investigators developed a CONSORT flow diagram and CONSORT checklist of the trial in accordance with the Consolidated Standards of Reporting Trials Statement (additional file1) [26]. The CONSORT flow diagram is described elsewhere [27]. The trial was registered in the Clinical Trials.gov (NCT03627156). The trial was registered after starting recruitment of the study participants due to network problems to collect data in non-fasting and non-feasting days because in Ethiopia there is fasting after Mid-August. The Institutional Review Board of Bahir Dar University gave an approval letter to conduct this trial (protocol number: 92/18-04). Before the implementation of the trial, each woman provided written informed consent (fingerprint for women who couldn’t read and write).

### Recruitment, randomization and intervention allocation

A randomized cluster sampling technique was used in this study. “*Kebele”* (the smallest administrative unit) was taken as a cluster. Cluster randomization was used to avoid counseling message contamination since women in the same cluster had a high probability of meeting and discussing nutrition messages. Moreover, to reduce the likelihood of communicating women in the intervention and control clusters, there were non-selected clusters (buffer zone) between the two groups [28]. From the seven woredas, three (*Bahir Dar Zuria*, *South Achefer*, and *Burie Zuria Woredas*) were selected by a simple random sampling (SRS) method.

**Fig 1:**
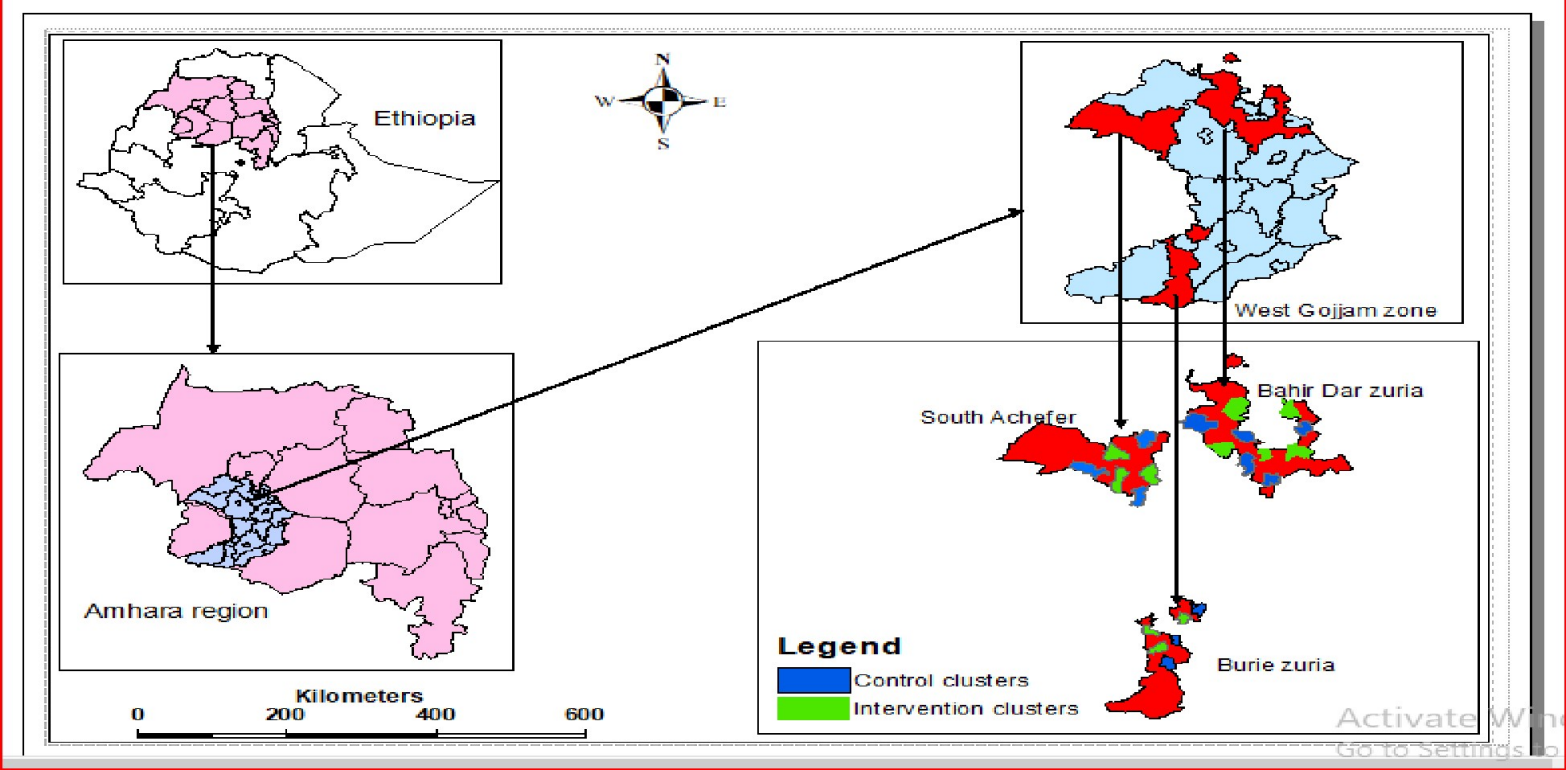
Study clusters in the study area.

Clusters (Kebele) were also selected using the SRS method. The numbers of selected clusters were ten in *Bahir Dar Zuria Woreda* and six clusters each in *South Achefer* and *Burie Zuria Woredas*. Lastly, an equal number of clusters in each woreda were randomly allocated as an intervention clusters and control clusters using the SRS (lottery) method (Fig 1). Eligible pregnant women were screened through the house to house survey using the first date of the last menstrual period and a pregnancy test. All pregnant women in selected clusters who fulfill the inclusion criteria were enrolled in the trial.

### Intervention

The intervention package for this study was community-based guided counseling using the HBM and the TPB. The counseling guide was prepared based on the recommendations of the World Health Organization and Ministry of Health of Ethiopia [29, 30]. Increasing meal frequency and portion size with increasing gestational age and taking diversified meals were the core contents of the counseling guide. Consumption of iron/folic acid supplementation, iodized salt use, reducing of a heavy workload, taking day rest, use of impregnated bed nets, and health services were also the key messages of the counseling guide.

**Fig 2:**
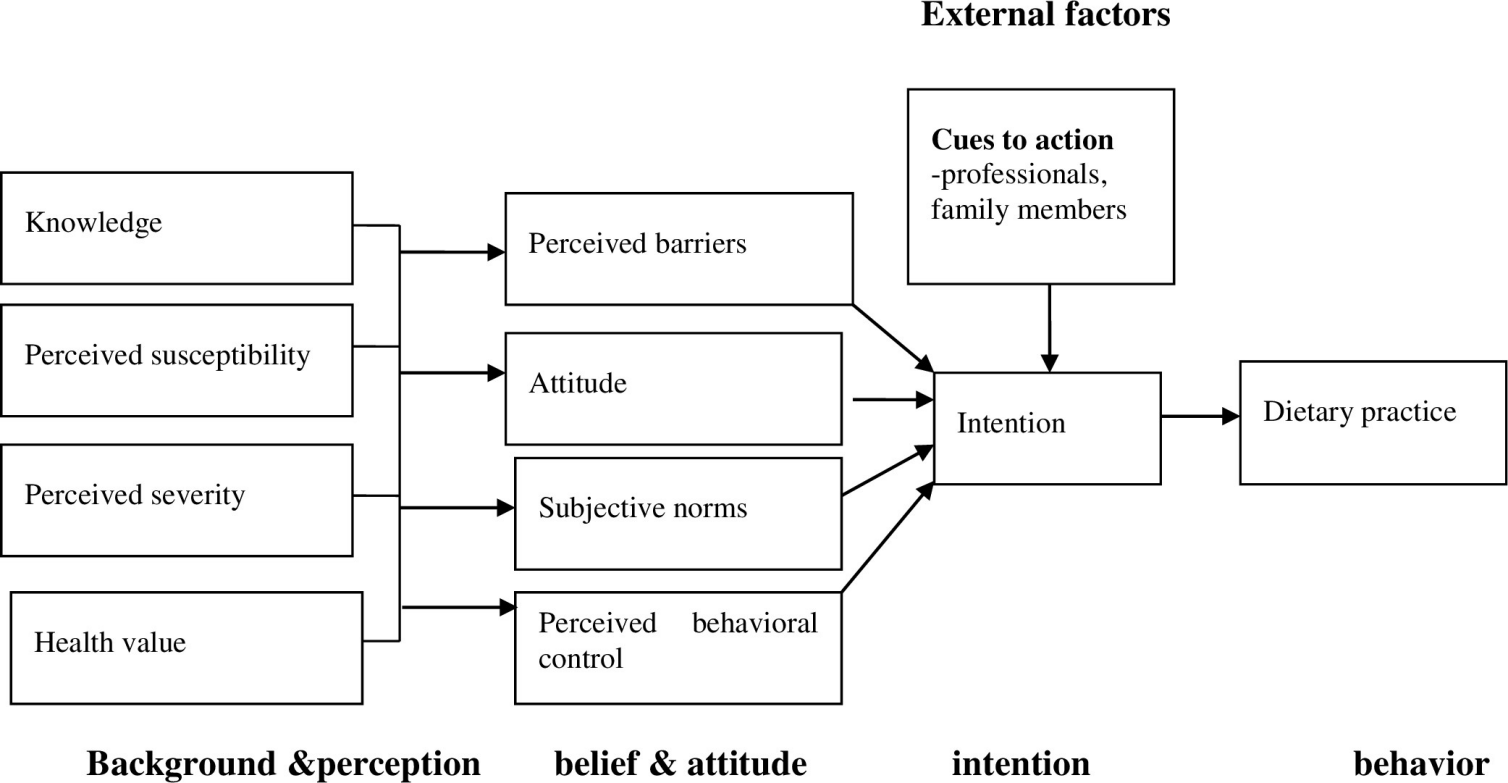
A new model: Combined health belief model and theory of planned behavior adopted from SUN X et al. [31].

The consequences of taking an inadequate amount and less diversified meal, susceptibility to and severity of the consequences of taking a less diversified meal, benefits of taking a healthy diet and barriers for taking a balanced diet were also discussed during counseling. The counselors assessed attitude, subjective norms, self-efficacy, perceived control, intention, knowledge, and dietary practices of women during each counseling session (Fig 2). Then, they counseled the women based on the identified gaps and women’s socioeconomic status.

Counseling was given monthly using a counseling guide with core contents. Individual nutrition counseling was given through a home visit on non-working days (religious holidays and weekends). Each counseling session lasted for 40 to 60 minutes. Each woman attended four counseling sessions throughout her entire pregnancy. During counseling, counselors used a client-centered approach to identify women’s dietary practices and their specific needs in terms of nutrition. Counselors first asked the women about their income, nutrients needed during pregnancy, and their dietary practices. Then, they considered women’s nutrient demand, income, and identified gaps. They allowed the women to choose from recommendations that were based on locally available, affordable and acceptable foodstuffs.

The first counseling was given before 16 weeks of gestation, focused on basic nutrition, food groups, food selection, preparation, meal frequency, portion size and iodized salt utilization. The second and third counseling sessions were given during the second trimester of pregnancy and covered the entire contents of the counseling guide. The last counseling was given based on the identified gaps during the early third trimester of pregnancy.

Leaflets with core messages in Amharic (local language) and appropriate pictures were prepared and delivered to each pregnant woman in the intervention group. Anyone at home or in the neighborhood who could read was suggested to read the leaflet for the woman who couldn’t read.

Women in the control group received nutrition education given by the health system. Pregnant women from both the control and intervention groups attended ANC services. Counseling and supervision of the counseling process were done by six BSc nurses and three MSc nutritionists, respectively. The counselors had previous experience of giving counseling services. A three-day intensive training was given to the counselors and supervisors using a counseling guide. The guide included ethical issues, maternal nutrition, HBM, and TPB constructs. It also covered the way of delivering nutrition counseling including role-playing and field practice. A one-day additional training was given to the counseling team after two months of intervention to maintain providers’ knowledge and counseling skills.

Fidelity of the intervention was checked using criteria that were developed by the investigators. The criteria were adopted from the National Institutes of Health Behavioral Change Consortium developed best practice recommendations [32]. The criteria included a checklist to assess intervention design, training of counselors, counseling process, receipt of intervention, and enactment of skills gained from the intervention [33].

### Data collection tools and procedures

Full details of the data collection tools and procedures were published previously [12]. Six nurses collected data using structured questionnaires through one-to-one interviews made with the participants at their homes. Data on socio-demographic and obstetric characteristics were collected at the baseline. Whereas, data on dietary practice, food security, HBM, and TPB constructs were taken before and after implementation of the trial.

The primary outcome of this trial was the dietary practice, which was assessed using a food frequency questionnaire (FFQ). Post-intervention data were collected between 36 to 37 weeks of gestation. Women who didn’t attend all counseling sessions were considered non-adherent to the guideline. But, women who withdraw from participating in the study were labeled as lost to follow up. The data collection team was trained for three days using a training manual. The data collection supervisors and the principal investigator closely followed the data collection process.

### Measurements

A food frequency questionnaire was taken from the literature and customized based on locally available foodstuffs [34]. The questionnaire contained 54 food items. When the women consumed a food item at least once a day, they categorized under the consumers’ category, otherwise not [35]. Food items of the FFQ were categorized into nine food groups [36]. Dietary diversity score (DDS) was analyzed by counting the number of food groups the women consumed within a week. The sum of food groups was divided into terciles (three parts) and women who took the highest tercile were considered having high DDS, or else not. Individual food items the women took within a week were counted and the mean food variety score (FVS) was analyzed. Women who scored above the mean FVS were labeled as having good FVS, otherwise not.

The frequencies of each animal source foods the women ate within the days of a week were counted to assess animal source food (ASF) consumption. Then, the frequency of ASF consumption was divided into three parts (tercile). Women who had the highest tercile were classified under high consumption of ASF. Women who took four or more meals a day were considered having adequate meal frequency, otherwise having inadequate meal frequency.

When women had high DDS, ASF consumption, FVS above the mean and adequate meal frequency, they were labeled as having appropriate dietary practice. Household food security was determined using 27 questions adapted from previously validated tools [37]. Food secure households did not report any food insecurity indicators except rarely worry due to fear of shortage of food in the household [37].

Principal Component Analysis (PCA) was done to assess the wealth index of the household. Quintiles of the wealth score were calculated based on the first principal component. Eight questions were used to determine women’s decision making power. Code one was given for each question when a decision was made by the woman alone or jointly with her husband otherwise zero [2]. Subjective norms, knowledge, attitude, intention, perceived susceptibility, perceived severity, perceived benefit, and perceived barriers were assessed using the sum of their respective composite questions.

### Data management and analysis

Data were entered into Epi info version 7.2. Software and exported to SPSS version 23 Software for analysis. The baseline difference in socio-demographic characteristics between the two groups was examined using a chi-square test. Pre- and post-intervention differences in dietary practice, DDS, FVS, ASF consumption, and meal frequency between the intervention and control groups were analyzed using a McNemar test.

Generalized Estimating Equation (GEE) with a binary logit function was used to examine the change difference of outcome between the intervention and control groups. GEE was run to accommodate clustered data and correlation of observations within subjects. The unstructured covariance matrix was considered during fitting the model while accounting for the effects of potential confounding factors. Crop production, socio-demographic variables, obstetric characteristics, food security, time, treatment, time and treatment interaction were analyzed. The effect of the intervention was assessed using time and treatment interaction. Odds ratios and respective 95% confidence intervals were calculated. *P* values < 0.05 were considered statistically significant.

## Results

### Socio-demographic characteristics of pregnant women

Seven hundred twelve pregnant women were recruited in this study. Among them, 694 respondents gave complete baseline data and were randomized into the intervention (346 women) and control (348 women) groups. At the end of this trial, 645 **(IG = 313, CG = 332)** respondents strictly adhered to the protocol. Baseline data on wealth index, occupation, age, ethnicity, family size, education, and religion of the respondents were similar between the two groups (P > 0.05). The socio-demographic characteristics of the pregnant women is available everywhere [27].

### Health belief model and theory of planned behavior constructs and their correlation with knowledge and dietary practice of pregnant women

Women in the intervention group demonstrated better scores of the HBM and the TPB constructs (P< 0.001) compared with women in the control group and their baseline scores. Comparison of the HBM and the TPB constructs score within and between intervention and control groups were published previously [27].

Except for the perceived barrier, the intervention had a strong positive correlation with all other HBM and TPB constructs (*P<0*.*001)*. All HBM and TPB constructs also showed a significant positive correlation with knowledge and the dietary practice of pregnant women (*P<0*.*001)*. Correlation of the HBM and the TPB constructs with knowledge and dietary practices of pregnant women were published previously [27].

### Effect of the intervention on the dietary practices of pregnant women

Before the implementation of the trial, there was no statistically significant difference in the dietary practice, DDS, ASF consumption, and frequency of meal between the two groups. Pregnant women in the control group had better FVS than women in the intervention group at the baseline (Table 1).

**Table 1:** Baseline dietary practices of pregnant women in West Gojjam Zone.

Variables	Intervention group(n_1_ = 313)	Control group (n_2_ = 332)	P
	Frequency (%)	Frequency (%)	
**Dietary practice**			
**Appropriate**	59(18.8)	74(22.3)	
**Inappropriate**	254(81.2)	258(77.7)	0.281
**DDS**			
**High**	98(31.3)	119(35.8)	
**Low**	215(68.7)	213(64.2)	0.223
**ASF consumption**			
**High**	109(34.8)	133(40.1)	
**Low**	204(65.2)	199(59.9)	0.098
**FVS**			
**Above the mean**	127(40.6)	176(53.0)	
**Mean and less**	186(59.4)	156(47.0)	0.002
**Frequency of meal**			
**More than three**	111(35.5)	123(37.0)	
**Three or less**	202(64.5)	209(63.0)	0.676

After the trial, women in the intervention group showed a significant improvement in the dietary practices, DDS, ASF consumption, FVS and frequency of meals compared with their dietary practices before the intervention. In the control group, the proportions of women who had appropriate dietary practices, high DDS, high ASF consumption, FVS above the mean and more than three meals showed a significant reduction at the end of the study.

At the end of this trial, the proportion of women who had appropriate dietary practices increased by 18.3% in the intervention group. However, the number of women who had appropriate dietary practices decreased by 12.4% in the control group. The average difference of appropriate dietary practice between the two groups was 30.7% (P<0.001).

Additionally, in the intervention group, DDS improved by 24.6%, whereas in the control group, it showed a 15.9% reduction, with an overall difference of 40.5% between the two groups (P<0.001). Similarly, FVS increased by 22.7% among women in the intervention group while it decreased by 13.2% in the control group, with an average difference of 35.9% between the two groups (P<0.001).

In the same way, the proportion of women who took ASF increased by 10.9% in the intervention group while it decreased by 9.7% in the control group. The overall difference in ASF consumption was 20.6% between the two groups (P<0.001). The proportion of women who took four or more meals increased by 38.0% in the intervention group, whereas it decreased by 9% in the control group. The overall difference in meal frequency was 47.0% between the two groups (P<0.001) (Table 2).

**Table 2:** Differences between baseline and end line dietary practices and difference of the differences between intervention and control groups.

	Intervention group(n = 313)			Control group(n = 332)			
Variable	Baseline	End line	Difference (EL-BL)	Baseline	End line	Difference (EL-BL)	Difference of difference
**Dietary practice**	0.188	0.371	0.183***	0.223	0.099	-0.124***	0.307***
**DDS**	0.313	0.559	0.246***	0.358	0.199	-0.159***	0.405***
**FVS**	0.406	0.633	0.227***	0.530	0.398	-0.132***	0.359***
**ASF**	0.348	0.457	0.109***	0.401	0.304	-0.097***	0.206***
**Meal frequency**	0.351	0.731	0.380***	0.370	0.280	-0.090***	0.470***

^*** P<0.001.^

On the multivariable Generalized Estimating Equations model, after controlling for possible confounders, pregnant women in the intervention group were 7.2 times more likely to have appropriate dietary practices compared to the control group [AOR = 7.187, 95% CI: (4.49, 11.49)]. Likewise, women in the intervention group were 7 times at higher odds of taking diversified meals than those in the control group [AOR = 6.994, 95% CI: (4.59, 10.66)].

Similarly, women who got counseling were 5 times [AOR = 4.928, 95% CI: (3.31, 7.33)] and 2.5 times [AOR = 2.531, 95% CI: (1.76, 3.63)] more likely to had better FVS and ASF consumption than women in the control group, respectively. Women in the intervention group were 8 times more likely to take four or more meals a day compared with women in the control group [AOR = 8.146, 95% CI: (5.377, 12.341)] (Table 3).

**Table 3:** GEE result of the intervention effect.

Variable		Beta coefficient	Standard error	95% CI	P	AOR	95% CI
**Dietary practice**	Intercept	-2.094	.6043	-3.278, -.909	.001	.123	.038, .403
	Time	-1.002	.1729	-1.341, -.663	< .001	.367	.262, .515
	Group	-.188	.1977	-.575, .200	.342	.829	.562, 1.221
	Time*group	1.974	.2414	1.501, 2.447	< .001	**7.200**	4.486, 11.556
**DDS**	Intercept	-.989	.5469	-2.061, .083	.071	.372	.127, 1.086
	Time	-.877	.1430	-1.157, -.596	< .001	.416	.314, .551
	Group	-.142	.1735	-.482, .198	.414	.868	.618, 1.219
	Time*group	1.975	.2174	1.549, 2.401	< .001	**7.209**	4.707, 11.039
**FVS**	Intercept	-.707	.4898	-1.667, .253	.149	.493	.189, -.707
	Time	-.589	.1250	-.834, -.344	< .001	.555	.434, -.589
	Group	-.454	.1647	-.777, -.131	.006	.635	.460, -.454
	Time*group	1.615	.2067	1.210, 2.020	< .001	**5.026**	3.352, 1.615
**ASF**	Intercept	-1.401	.5112	-2.403, -.399	.006	.246	.090,.671
	Time	-.452	.1252	-.698, -.207	< .001	.636	.498, .813
	Group	-.202	.1673	-.530, .126	.226	.817	.588, 1.134
	Time*group	.927	.1867	.561, 1.293	< .001	**2.527**	1.753, 3.643
**Meal frequency**	Intercept	-1.037	.5065	-2.030, -.044	.041	.355	.131, .957
	Time	-.448	.1358	-.714, -.182	.001	.639	.490, .834
	Group	.022	.1686	-.308, .353	.894	1.023	.735, 1.423
	Time*group	2.156	.2165	1.731, 2.580	< .001	**8.634**	5.648, 13.198

The model was adjusted for wealth index, food security, decision making power, education, age, parity, gravidity and family size

AOR = adjusted odds ratio, CI = confidence interval, P = P-value.

## Discussion

In this study, nutrition counseling using the HBM and the TPB constructs was effective on improving the dietary practices of pregnant women. The proportion of women who had appropriate dietary practices was significantly higher in the intervention group compared with women in the control group. This finding persists after controlling the potential confounders.

This result is in agreement with the report of a study from Dessie Town which reported significant improvement in the dietary practices of pregnant women who attended nutrition education using the HBM [10]. A similar positive finding was also reported from the Alaje district of northern Ethiopia, Addis Ababa, Burkina Faso, and Iran that showed the positive effects of nutrition counseling on improving the dietary practices of pregnant women [38–41].

The possible explanation for the success of this intervention might be the method of counseling. In this study, counseling was given using a counseling guide and simple messages with culturally appropriate pictures. Counseling was delivered using a client-centered two-way communication approach. The counselors used trimester based education. Besides, before each counseling session, counselors assessed existing knowledge, dietary practice and socio-economic situation of each woman. Then, counseling was given based on the need of specific women. All these encourage behavior change towards having appropriate dietary habit. Moreover, this study used leaflets with core messages and appropriate pictures, which might help women in the intervention group to remember key messages.

Furthermore, in this study counseling was given using the HBM and the TPB constructs. Scholars who used the HBM and the TPB for counseling pregnant women have also reported a positive result on improving the dietary practices of pregnant women [10]. This might be due to the fact that counseled women perceived that poor diet during pregnancy had severe adverse effects on them and their fetus. They considered themselves to be at risk to the negative consequences of an unhealthy diet. They perceived taking a healthy diet is crucial to maternal and fetal health. All this motivated them to take action towards improving their nutrient intakes.

Additionally, counseling increased perceived behavioral control, self-efficacy, knowledge, and attitudes regarding having a healthy diet, which in turn improved the intention of having appropriate dietary practice. The intention is the direct determinant of having healthy dietary practice.

Nutrient requirements increase during pregnancy and with increasing gestational age. However, most Ethiopian women perceived that increasing nutrient intake while they are pregnant leads to having a big baby that further predispose to difficulty during delivery. As a result, they practice eating less while they are pregnant compared with their practices before pregnancy [5, 11, 42].

Moreover, the dietary practices of pregnant women decrease with increasing gestational age. According to Kuche D *et al*, nutrient intakes of the third trimester pregnant women were less than nutrient intakes of women before the third trimester [43]. In line with the report of previous researchers, in this study, dietary practices of pregnant women in the control group decreased from their baseline dietary practices.

The possible justification for the inappropriate dietary practices of pregnant women in the control group was poor counseling practice of the health workers. During nutrition education, professionals don’t use counseling guide and behavioral models. They advise pregnant women to take one additional meal from foods available at home compared to their meals before pregnancy [12]. Thus, women may not understand the type and amount of food needed for them. This type of simple advice never brings behavior change towards healthy eating habits.

The second possible justification may be the study setting. This study was done among women in rural settings. Women in rural areas were more likely to practice eating down due to the high prevalence of food taboo in the rural areas than in urban settings [6]. Seasonal variation might be the other explanation for reducing the dietary intake of women in the control group. In both groups, baseline quantitative data from the majority of the study participants were collected from May to June, whereas end line data were collected from September to October.

The study area is extremely dependent on rain-fed harvesting or the “Meher” harvest. Harvest season starts in November and continuous until February. Therefore, the availability of crops in the household deteriorates during the lean season, particularly between July and October. This may be the reason for the lower proportion of the appropriate dietary practices of pregnant women.

Women in the intervention group had higher dietary diversity and food variety scores compared with the control group. The reason for this might be due to the use of a counseling manual with a detail description of each foodstuff with its benefits for the women and the growing fetus. The positive effect of counseling on diet during pregnancy on improving dietary diversity was reported by previous studies. Studies done in Burkina Faso and Iran showed that the dietary diversity of pregnant women in the intervention group showed a significant improvement compared with the control group [40, 44]. A study done among under two years old children also reported a significant improvement in the dietary diversity of children in the intervention group after nutrition education intervention [45].

Women who attended guided counseling reported improvement in animal source food consumption than women who attended nutrition education given by the health system. This finding is in agreement with the previous study done among under two years old children that reported significant improvement of ASF consumption in the intervention group compared to children in the control group [45]. One of the core contents of the counseling guide was taking animal products three times a day and its importance during pregnancy. This might increase women’s knowledge and commitment to take ASFs.

The likelihood of taking four or more meals a day was higher among women in the intervention group compared with women in the control group. The previous study that assessed the effect of nutrition education on infant feeding practice showed a significant improvement in the frequency of meal in the intervention group [45]. This might also be due to the counseling method as increasing frequency of meals and portion sizes with increased gestational age were the two core contents of the counseling guide. Accordingly, counselors gave emphasis during counseling for the frequency of meals and portion size during each counseling session. The observed positive effects on dietary practices confirm that dietary practices of pregnant women can be modified through community-based guided counseling.

Implication: The findings of this study have significant practical implications for improving nutrition counseling methods, which in turn will improve maternal and child health. The fact that significantly higher numbers of pregnant women have appropriate dietary practices implies the need for enacting the community level education through health extension workers using theory enhanced approaches.

Being a randomized controlled trial using a client-centered approach and trimester based counseling were the strengths of this study. However, this study has some limitations. All responses were self-reported which were dependent on the women’s memory and honesty in answering questions. The post-intervention result may not have lasted longer since it was a relatively short term intervention.

## Conclusion

Counseling using the health belief model and the theory of planned behavior is an effective approach in increasing the proportion of women who had appropriate dietary practices. Women in the intervention group had high DDS, FVS above the mean, high ASF consumption, and adequate food frequency than women in the control group. Therefore, the findings of this study suggest the need for employing trimester based counseling using the HBM and the TPB to improve dietary practices of pregnant women. Moreover, this study recommends developing nutrition counseling guidelines with a detail description of diet during pregnancy, the HBM, and the TPB constructs.

## Supporting information

S1 Checklist (DOCX)

S1 Data (DOCX)

## Abbreviations

ANC: Antenatal Care
ASF: Animal Source Food
DDS: Dietary Diversity Score
FFQ: Food Frequency Questionnaire
FVS: Food Variety Score
HBM: Health Belief Model
LBW: Low Birth Weight
PCA: Principal Component Analysis
TPB: Theory of Planned Behavior
WHO: World Health Organization

## References

[1] Black RE, Victora CG, Walker SP, Bhutta ZqA, Christian P, et al (2013) Maternal and child undernutrition and overweight in low-income and middle-income countries. Lancet 382: 427–451. 10.1016/S0140-6736(13)60937-X 23746772

[2] Central Statistical Agency (CSA) [Ethiopia] and ICF (2016) Ethiopia Demographic and Health Survey 2016. Addis Ababa, Ethiopia, and Rockville, Maryland, USA: CSA and ICF.

[3] Zerfu TA, Biadgilign S (2018) Pregnant mothers have limited knowledge and poor dietary diversity practices, but favorable attitude towards nutritional recommendations in rural Ethiopia: evidence from community-based study. BMC Nutrition 4: 1–9. 10.1186/s40795-017-0207-6 32153904 PMC7050941

[4] Nana A, Zema T (2018) Dietary practices and associated factors during pregnancy in northwestern Ethiopia. BMC Pregnancy and Childbirth 18: 183 10.1186/s12884-018-1822-1 29801471 PMC5970492

[5] Asayehu TT, Lacha C, DeHenauw S, Gebreyesus SH (2016) Dietary behaviour, food and nutrient intake of women do not change during pregnancy in Southern Ethiopia. Maternal & Child Nutr 13: 10.1111/mcn.12343 27373896 PMC6866045

[6] Zerfu TA, Umeta M, Baye K (2016) Dietary habits, food taboos, and perceptions towards weight gain during pregnancy in Arsi, rural central Ethiopia: a qualitative cross-sectional study. Journal of Health, Population and Nutrition 35: 22: 10.1186/s41043-41016-40059-41048 PMC5025964 27456151

[7] Tenaw Z, Arega M, Tachbele E (2018) Nutritional knowledge, attitude and practices among pregnant women who attend antenatal care at public hospitals of Addis Ababa, Ethiopia. Int J Nurs Midwifery 10: 81–89.

[8] Hambidge KM, Krebs NF (2018) Strategies for optimizing maternal nutrition to promote infant development. Reproductive Health 15: 87: 10.1186/s12978-018-0534-3 29945648 PMC6019994

[9] Federal Democratic Republic of Ethiopia (2016) National Guideline on Adolescent,Maternal Infant and Young Child Nutrition. Ethiopia

[10] Diddana TZ, Kelkay GN, Dola AN, Sadore AA (2018) Effect of Nutrition Education Based on Health Belief Model on Nutritional Knowledge and Dietary Practice of Pregnant Women in Dessie Town, Northeast Ethiopia: A Cluster Randomized Control Trial. Journal of Nutrition and Metabolism 2018. 10.1155/2018/6731815 PMC6033240 30034866

[11] Saldanha LS, Buback L, White JM, Mulugeta A, Mariam SG, et al (2012) Policies and program implementation experience to improve maternal nutrition in Ethiopia. Food and Nutrition Bulletin 33: S27–S50. 10.1177/15648265120332S103 22913106

[12] Demilew YM, Alene GD, Belachew T (2020) Dietary practices and associated factors among pregnant women in West Gojjam Zone, Northwest Ethiopia. BMC Pregnancy and Childbirth 20: 10.1186/s12884-12019-12702-z. PMC6945405 31906981

[13] FHI360 Behavior change communication. Academy for International Development. 2012, Available at: www.globalhealthcommunication.org/strategies/behavior_change_communication (access date October 10,2019).

[14] Sukhwinder K (2010) Outcomes of prenatal nutrition counseling In developing countries, A Literature Review.

[15] Spahn JM, Reeves RS, Keim KS, Laquatra I, Kellogg M, et al (2010) State of the Evidence Regarding Behavior Change Theories and Strategies in Nutrition Counseling to Facilitate Health and Food Behavior Change. J Am Diet Assoc 110: 879–891. 10.1016/j.jada.2010.03.021 20497777

[16] Salama AM (2018) Utilizing Health Belief Model to Enhance the Preventive Behavior against Iron-Deficiency Anemia among Pregnant Women. IOSR Journal of Nursing and Health Science 7: 59–69.

[17] Aşcı Ö, Rathfisch G (2016) Effect of lifestyle interventions of pregnant women on their dietary habits, lifestyle behaviors, and weight gain: a randomized controlled trial. Aşcı and Rathfisch Journal of Health, Population and Nutrition 35. 10.1186/s41043-016-0044-2 PMC5025976 26911204

[18] Williams L, Campbell K, Abbott G, Crawford D, Ball K (2012) Is maternal nutrition knowledge more strongly associated with the diets of mothers or their school-aged children?. Public Health Nutr 15: 1396–1401. 10.1017/S1368980011003430 22230490

[19] Sobal J, Bisogni CA (2009) Constructing Food Choice Decisions. ann behav med 38: S37–S46. 10.1007/s12160-009-9124-5 19787306

[20] Glanz K, Rimer BK, Viswanath K, editors (2008) Health behavior and health education: theory, research, and practice /. 4th ed San Francisco: Jossey-Bass.

[21] Jones CL, Jensen JD, Scherr CL, Brown NR, Christy K, et al (2015) The Health Belief Model as an Explanatory Framework in Communication Research: Exploring Parallel, Serial, and Moderated Mediation. Health Commun 30: 566–576. 10.1080/10410236.2013.873363 25010519 PMC4530978

[22] Ajzen I (1991) The theory of planned behavior. Organizational Behavior and Human Decision Processes 50: 179–211:doi: 110.1016/0749-5978(1091)90020-T.

[23] Asare M (2015) Using the Theory of Planned Behavior to Determine the Condom Use Behavior among College Students. Am J Health Stud 30: 43–50. 26512197 PMC4621079

[24] World Health Organization (2001) World Medical Association Declaration of Helsinki. Ethical Principles for Medical Research Involving Human Subjects. Bulletin of the World Health Organization 79: 373–374. 11357217 PMC2566407

[25] West KP Jr, Christian P, Labrique AB, Rashid M, Shamim AA, et al (2011) Effects of Vitamin A or Beta Carotene SupplementationonPregnancy-RelatedMortality and Infant Mortality in Rural Bangladesh. A Cluster Randomized Trial. JAMA 305: 1986–1995. 10.1001/jama.2011.656 21586714

[26] Schulz KF, Altman DG, Moher D (2010) CONSORORT 2010 Statement: updated guidelines for reporting parallel group randomised trials. BMJ 340: c332: 10.1136/bmj.c332 20332509 PMC2844940

[27] Demilew YM, Alene GD, Belachew T (2020) Effect of guided counseling on nutritional status of pregnant women in West Gojjam zone, Ethiopia: a cluster-randomized controlled trial. Nutrition Journal 19: 1–12. 10.1186/s12937-019-0518-3 32345286 PMC7189500

[28] Chowdhury M, Raynes-Greenow C, Alam A, Dibley MJ (2017) Making a balanced plate for pregnant women to improve birthweight of infants: a study protocol for a cluster randomised controlled trial in rural Bangladesh. BMJ Open 7 e015393 10.1136/bmjopen-2016-015393 28827238 PMC5724074

[29] Federal Democratic Republic of Ethiopia (2013) Training of Trainers Manual for Counseling on Maternal, Infant, and Young Child Nutrition Ethiopia: http://iycn.wpengine.netdna-cdn.com/files/IYCN_MIYCN_Ethiopia_Counseling_TOT_Manual_1211.pdf (access date March 9,2020).

[30] UNICEF (2012) The Community Infant and Young Child Feeding Counseling Package: Key Messages Booklet. https://www.unicef.org/nutrition/files/Key_Message_Booklet_2012_small.pdf (access date March 9,2020).

[31] Sun X, Guo Y, Wang S, Sun J (2006) Predicting Iron-Fortified Soy Sauce Consumption Intention: Application of the Theory of Planned Behavior and Health Belief Model. J Nutr Educ Behav 38: 276–285. 10.1016/j.jneb.2006.04.144 16966048

[32] Bellg AJ BB, Resnick B, Hecht J, Minicucci DS, Ory M, et al (2004) Enhancing treatment fidelity in health behavior change studies: best practices and recommendations from the NIH Behavior Change Consortium. Health Psychol 23: 443–451. 10.1037/0278-6133.23.5.443 15367063

[33] Borrelli B (2011) The Assessment, Monitoring, and Enhancement of Treatment Fidelity In Public Health Clinical Trials. J Public Health Dent 71: S52–S63. 10.1111/j.1752-7325.2011.00233.x 21499543 PMC3074245

[34] Ethiopian Public Health Institute (2013) Ethiopia National Food Consumption Survey. Addis Ababa: EPHI.

[35] Workicho A, Belachew T, Feyissa GT, Wondafrash B, Lachat C, et al (2016) Household dietary diversity and Animal Source Food consumption in Ethiopia: evidence from the 2011 Welfare Monitoring Survey. BMC Public Health 16: 1192 10.1186/s12889-016-3861-8 27884138 PMC5123272

[36] FAO and FHI 360 (2016) Minimum Dietary Diversity for Women: A Guide for Measurement. Rome: FAO.

[37] Gebreyesus SH, Lunde T, Mariam DH, Woldehanna T, Lindtjørn B (2015) Is the adapted Household Food Insecurity Access Scale (HFIAS) developed internationally to measure food insecurity valid in urban and rural households of Ethiopia?. BMC Nutrition 1: http://www.biomedcentral.com/bmcnutr/content/1//2.

[38] Zelalem A, Endeshaw M, Ayenew M, Shiferaw S, Yirgu R (2017) Effect of nutrition education on pregnancy specific nutrition knowledge and healthy dietary practice among pregnant women in Addis Ababa. Clinics in Mother and Child Health 14: 265.

[39] Selvakumar DL (2015) Relationships between a Prenatal Nutrition Education Intervention and Maternal Nutrition in Ethiopia. BMJ Open 5: A1–A53.

[40] Nikièma L, Huybregts L, Martin-Prevel Y, Donnen P, Lanou H, et al (2017) Effectiveness of facility-based personalized maternal nutrition counseling in improving child growth and morbidity up to 18 months: A cluster-randomized controlled trial in rural Burkina Faso. PLoS ONE 12: e0177839 10.1371/journal.pone.0177839 28542391 PMC5444625

[41] Khoramabadi M., Dolatian M., Hajian S., Zamanian M., Taheripanah R., et al (2016) Effects of Education Based on Health Belief Model on Dietary Behaviors of Iranian Pregnant Women. Global Journal of Health Science 8: 230–239. 10.5539/gjhs.v8n2p230 PMC4803956 26383208

[42] Hadush Z, Birhanu Z, Chaka M, Gebreyesus H (2017) Foods tabooed for pregnant women in Abala district of Afar region, Ethiopia: an inductive qualitative study. BMC Nutrition 3: 40: 10.1186/s40795-017-0159-x 32153820 PMC7050739

[43] Kuche D, Singh P, Moges D, Belachew T (2015) Nutritional Status and Associated Factors among Pregnant Women in Wondo Genet District, Southern Ethiopia. Journal of Food Science and Engineering 5: 85–94 10.17265/12159-15828/12015.17202.17005

[44] Mohajeri M, Barzegar A, Nemati A, Rafati P (2018) Can nutrition education improve nutritional status in pregnant women? Nutrafoods 17: 23–26: 10.17470/NF-17017-11011-17471

[45] Kuchenbecker J, Reinbott A, Mtimuni B, Krawinkel MB, Jordan I (2017) Nutrition education improves dietary diversity of children 6–23 months at community-level: Results from a cluster randomized controlled trial in Malawi. PLoS ONE 12: e0175216 10.1371/journal.pone.0175216 28426678 PMC5398527

