# Supplementary material for: Effect of guided counseling on dietary practices of pregnant women in West Gojjam Zone, Ethiopia
Source: PLoS One. 2020 May 26;15(5):e0233429. doi: 10.1371/journal.pone.0233429 (PMC7250435; doi:10.1371/journal.pone.0233429)
Supplement: S1 Data — (DOCX) [file pone.0233429.s002.docx]

### Annex IV: Structured English questionnaire

Date of the interview-------------------------------

Interviewer Id No -------------------------------

Time of starting interview--------------------

Time of completing interview-------------------

Name of the cluster ----------------------------

Instruction – indicate the answer by encircle the number which contains the correct answer or write the correct answer in the blank space.

Table 16: Socio-demographic related questions

| **S.No** | **Variable** | | **Response** | | | | | | | |
| --- | --- | --- | --- | --- | --- | --- | --- | --- | --- | --- |
|  | **Did you have known medical or surgical illness? If no start interview** | | | | | | | | | |
| 101 | How old are you?(probe for best estimate) | ---------years | | | |  | | |  | |
| 102 | What is your religion? | 1. Orthodox  2. Protestant | | | | 3. Muslim  4. Catholic | | | 5.others.specify --- | |
| 103 | Which ethnic group do you belong to? | 1. Amhara  3.Tigrie | | | | 2. Agew  4. Oromo | | | 5.others.Specify--- | |
| 104 | What is the highest education level you completed? | 1.Can’t read&write  2.Can read &write | | | | 3.Primary  4.Secondary | | | 5.College & above | |
| 105 | What is your current occupation? | 1.House wife  2.Daily laborer | | | | 3.Farmer  4.G/ employee | | | 5.Merchant  6.Other.specify--- | |
| 106 | How many is your family size (Total number of family members)? | In number ------ | | | |  | | |  | |
| 107 | What is your current marital status? | 1.Married  2.Single | | | | 3.Widowed  4.Divorced | | | 5.Other,specify--- | |
| 108 | What is the highest education level your husband completed? | 1.Can’t read& write  2.Can read &write | | | | 3.Primary  4.Secondary | | | 5.College & above | |
| 109 | What is your husband’s current occupation? | 1.P/employee  2.Daily laborer | | | | 3.Farmer  4.G/ employee | | | 5.Merchant  6.Other,specify--- | |
| 110 | Who is the head of the household? | 1.Husband only | | | | 2.Woman only | | | 3. Both | |
| Table 17:Household Variables | | | | | | | | | | |
| **S.No** | **Variable** | | | **Response** | | | | | | **Skip** |
| 201 | What type of house does this household have? | | | 1.Corrugated iron sheet house  2.Grass roof house | | | | | |  |
| 202 | Do you have separate room for sleeping? | | | 1. Yes | 2. No | |  | | | If no go to Q204 |
| 203 | If yes for Q202, how many rooms? | | | 1.One | 2.Two | | 3.Three | | |  |
| 204 | Do you have a separate room for animals? | | | 1. Yes | 2. No | |  | | |  |
| 205 | Do you have a separate room which is used as a kitchen? | | | 1. Yes | 2. No | |  | | |  |
| 206 | Does any member of this household own cows? | | | 1. Yes | 2. No | | If yes, how many? | | |  |
| 207 | Does any member of this household own oxen? | | | 1. Yes | 2. No | | If yes, how many? | | |  |
| 208 | Does any member of this household own calves? | | | 1. Yes | 2. No | | If yes, how many? | | |  |
| 209 | Does any member of this household own other cattle? | | | 1. Yes | 2. No | | If yes, how many? | | |  |
| 210 | Does any member of this household own horses? | | | 1. Yes | 2. No | | If yes, how many? | | |  |
| 211 | Does any member of this household own donkeys? | | | 1. Yes | 2. No | | If yes, how many? | | |  |
| 212 | Does any member of this household own mules? | | | 1. Yes | 2. No | | If yes, how many? | | |  |
| 213 | Does any member of this household own sheeps? | | | 1. Yes | 2. No | | If yes, how many? | | |  |
| 214 | Does any member of this household own goats? | | | 1. Yes | 2. No | | If yes, how many? | | |  |
| 215 | Does any member of this household own chickens? | | | 1. Yes | 2. No | | If yes, how many? | | |  |
| 216 | Does any member of this household own beehives? | | | 1. Yes | 2. No | | If yes, how many? | | |  |
| 217 | Does any member of this household own any agricultural land? | | | 1. Yes | 2. No | | If yes, how many hectare? | | |  |
| 218 | Does any member of this household own Radio? | | | 1. Yes | 2. No | | If yes, how many? | | |  |
| 219 | Does any member of this household own Television? | | | 1. Yes | 2. No | | If yes, how many? | | |  |
| 220 | Does any member of this household own Telephone (Mobile)? | | | 1. Yes | 2. No | | If yes, how many? | | |  |
| 221 | Does any member of this household own table? | | | 1. Yes | 2. No | | If yes, how many? | | |  |
| 222 | Does any member of this household own chair? | | | 1. Yes | 2. No | | If yes, how many? | | |  |
| 223 | Does any member of this household own bed with cotton/sponge/spring mattress? | | | 1. Yes | 2. No | | If yes, how many? | | |  |
| 224 | Does any member of this household own a solar lamp? | | | 1. Yes | 2. No | | If yes, how many? | | |  |
| 225 | Does any member of this household own watch? | | | 1. Yes | 2. No | | If yes, how many? | | |  |
| 226 | Does any member of this household own bicycle? | | | 1. Yes | 2. No | | If yes, how many? | | |  |
| 227 | Does any member of this household own motor? | | | 1. Yes | 2. No | | If yes, how many? | | |  |
| 228 | Does any member of this household own animal-drawn cart? | | | 1. Yes | 2. No | | If yes, how many? | | |  |
| 229 | Does any member of this household have a bank account? | | | 1. Yes | 2. No | | If yes, how many? | | |  |
| 230 | Does any member of this household own khat farm? | | | 1.Yes 2.No | If yes, how many hectare? | | | | |  |
| 231 | Does any member of this household own eucalyptus farm? | | | 1.Yes 2.No | If yes, how many hectare? | | | | |  |
| 232 | How many quintals of the following cereals do the family produced in this year? | | | 1.Teff--  2.Millet-  3.Maize- | 4.Wheat—  5.Barley--  6.Rye - | | | 7.Rice  8.Other, specify | |  |
| 233 | How many quintals of the following legume do the family produced in this year? | | | 1.Bean--  2.Pea-- | 3. Lentil-  4. Nut - | | | 5. Chick pea--  6. Grass pea-  7.Other specify-- | |  |
| 234 | How many kilograms of vegetables do the family produced? | | | 1.Gomen  2.Carrot  3. Potato | 4. Tomato  5. Cabbage  6. Onion  10. Garlic | | | 7.Beat root  8. Pumpkin  9.Pepper  11.Other specify | |  |
| 235 | How many kilograms of fruits do the family produced? | | | 1.Mango 2.Banana  3.Avocado | 4. Papaya  5. Guava  6.Lemon | | | 7. Other specify- | |  |

Table 18: Obstetric and medical history

| S.No | **Variable** | **Response** | **Skip** |
| --- | --- | --- | --- |
| 301 | How many times have you been pregnant so far? | In number ------ |  |
| 302 | How many times did you give birth so far? | In number ------- |  |
| 303 | Did you have plan to the current pregnancy? | 1.Yes 2.No |  |
| 304 | Did you have ANC visit to the current pregnancy? | 1.Yes 2.No | If no for Q304 go to Q307 |
| 305 | If yes for Q304, when did you start ANC visit? | --------weeks |  |
| 306 | If yes for Q304, how many visits do you have so far? | ------- |  |
| 307 | Do you have nausea during pregnancy? | 1. Yes, how sever is it? 2.No |  |
| 308 | Do you have vomiting during pregnancy? | 1. Yes, how sever is it? 2.No |  |
| 309 | Do you have any illness in the past two weeks? | 1.Yes 2.No |  |
| 310 | If yes for Q309. Which symptom did/do you have? |  | If no for Q309 go to Q312 |
| 311 | If yes for Q309. What measure did you take to manage it? | --------- |  |
| 312 | Did/do you take Iron/folic acid tablet? | 1.Yes 2.No |  |
| 313 | If yes for Q312, when you started to take? | ------weeks | If no for Q312 go to Q316 |
| 314 | If yes for Q312, how often? | --day,--/week,--/month |  |
| 315 | If yes for Q312, how long did you take it? | ------------ |  |
| 316 | For woman who did not take iron/folic acid tablet daily, what could be the reason for refraining from taking it? | ------------------- |  |
| 317 | Have you got counseling on maternal nutrition during this pregnancy? | 1.Yes | 2.No |
| 318 | If yes for Q317, who provide nutrition counseling? | 1.HP 2. HEWs 3.Other | If no for Q317 go to Q319 |
| 319 | Distance of the nearby health institution from your home is? | ----- |  |

Table 19: Dietary practice of the pregnant women

**Ask the respondent if a food item from the following food groups was consumed in the form of injera, porridge, bread, soup, juice and roast etc in the previous day, last week or three months.**

| **S.No** | **Variable** | **Response** | |
| --- | --- | --- | --- |
|  |  | **If yes for the following questions, how often per day or per week or per month were followed to each question whereas if the response is no go to the next question** | |
| 501 | Did you take teff in the last three months? | 1.Yes 2.No | Per day--week--month -- |
| 502 | Did you take dagusa (millet) in the last three months? | 1.Yes 2.No | Per day--week--month -- |
| 503 | Did you take maize in the last three months? | 1.Yes 2.No | Per day--week--month -- |
| 504 | Did you take wheat in the last three months? | 1.Yes 2.No | Per day--week--month -- |
| 505 | Did you take barley in the last three months? | 1.Yes 2.No | Per day--week--month -- |
| 506 | Did you take sorghum in the last three months? | 1.Yes 2.No | Per day--week--month -- |
| 507 | Did you take rice in the last three months? | 1.Yes 2.No | Per day--week--month -- |
| 508 | Did you take bean in the last three months? | 1.Yes 2.No | Per day--week--month -- |
| 509 | Did you take pea in the last three months? | 1.Yes 2.No | Per day--week--month -- |
| 510 | Did you take soya bean in the last three months? | 1.Yes 2.No | Per day--week--month -- |
| 511 | Did you take lentil in the last three months? | 1.Yes 2.No | Per day--week--month -- |
| 512 | Did you take kidney bean in the last three months? | 1.Yes 2.No | Per day--week--month -- |
| 513 | Did you take chick pea in the last three months? | 1.Yes 2.No | Per day--week--month -- |
| 514 | Did you take grass pea in the last three months? | 1.Yes 2.No | Per day--week--month -- |
| 515 | Did you take nut in the last three months? | 1.Yes 2.No | Per day--week--month -- |
| 516 | Did you take lupine in the last three months? | 1.Yes 2.No | Per day--week--month -- |
| 517 | Did you take sunflower seed in the last three months? | 1.Yes 2.No | Per day--week--month -- |
| 518 | Did you take linseed in the last three months? | 1.Yes 2.No | Per day--week--month -- |
| 519 | Did you take niger seed in the last three months? | 1.Yes 2.No | Per day--week--month -- |
| 520 | Did you take sesame seed in the last three months? | 1.Yes 2.No | Per day--week--month -- |
| 521 | Did you take potato in the last three months? | 1.Yes 2.No | Per day--week--month -- |
| 522 | Did you take sweet potato in the last three months? | 1.Yes 2.No | Per day--week--month -- |
| 523 | Did you take beetroot in the last three months? | 1.Yes 2.No | Per day--week--month -- |
| 524 | Did you take Ethiopian collard greens (gomen) in the last three months? | 1.Yes 2.No | Per day--week--month -- |
| 525 | Did you take cabbage in the last three months? | 1.Yes 2.No | Per day--week--month -- |
| 526 | Did you take carrot in the last three months? | 1.Yes 2.No | Per day--week--month -- |
| 527 | Did you take tomato in the last three months? | 1.Yes 2.No | Per day--week--month -- |
| 528 | Did you take salad in the last three months? | 1.Yes 2.No | Per day--week--month -- |
| 529 | Did you take pampkin in the last three months? | 1.Yes 2.No | Per day--week--month -- |
| 530 | Did you take green pepper in the last three months? | 1.Yes 2.No | Per day--week--month -- |
| 531 | Did you take meat in the last three months? | 1.Yes 2.No | Per day--week--month -- |
| 532 | Did you take liver in the last three months? | 1.Yes 2.No | Per day--week--month -- |
| 533 | Did you take kidney in the last three months? | 1.Yes 2.No | Per day--week--month -- |
| 534 | Did you take heart in the last three months? | 1.Yes 2.No | Per day--week--month -- |
| 535 | Did you take chicken in the last three months? | 1.Yes 2.No | Per day--week--month -- |
| 536 | Did you take egg in the last three months? | 1.Yes 2.No | Per day--week--month -- |
| 537 | Did you take milk in the last three months? | 1.Yes 2.No | Per day--week--month -- |
| 538 | Did you take cheese in the last three months? | 1.Yes 2.No | Per day--week--month -- |
| 539 | Did you take yogurt in the last three months? | 1.Yes 2.No | Per day--week--month -- |
| 540 | Did you take whey in the last three months? | 1.Yes 2.No | Per day--week--month -- |
| 541 | Did you take fish in the last three months? | 1.Yes 2.No | Per day--week--month -- |
| 542 | Did you take banana in the last three months? | 1.Yes 2.No | Per day--week--month -- |
| 543 | Did you take orange in the last three months? | 1.Yes 2.No | Per day--week--month -- |
| 544 | Did you take papaya in the last three months? | 1.Yes 2.No | Per day--week--month -- |
| 545 | Did you take lemon in the last three months? | 1.Yes 2.No | Per day--week--month -- |
| 546 | Did you take avocado in the last three months? | 1.Yes 2.No | Per day--week--month -- |
| 547 | Did you take guava in the last three months? | 1.Yes 2.No | Per day--week--month -- |
| 548 | Did you take mango in the last three months? | 1.Yes 2.No | Per day--week--month -- |
| 549 | Did you take wild fruit in the last three months? | 1.Yes 2.No | Per day--week--month--- specify------------ |
| 550 | Did you take pepper in the last three months? | 1.Yes 2.No | Per day--week--month -- |
| 551 | Did you take onion in the last three months? | 1.Yes 2.No | Per day--week--month -- |
| 552 | Did you take garlic in the last three months? | 1.Yes 2.No | Per day--week--month -- |
| 553 | Did you take iodized salt in the last three months? | 1.Yes 2.No | Per day--week--month -- |
| 554 | Did you take oil in the last three months? | 1.Yes 2.No | Per day--week--month -- |
| 555 | Did you take fat in the last three months? | 1.Yes 2.No | Per day--week--month -- |
| 556 | Did you take butter in the last three months? | 1.Yes 2.No | Per day--week--month -- |
| 557 | Did you take honey in the last three months? | 1.Yes 2.No | Per day--week--month -- |
| 558 | Did you take soft drink in the last three months? | 1.Yes 2.No | Per day--week--month--- |
| 559 | Did you take tea in the last three months? | 1.Yes 2.No | Per day--week--month -- |
| 560 | Did you take alcoholic beverages in the last three months? | 1.Yes 2.No | Per day--week--month -- |
| 561 | Which alcohol? | 1.Tella---- | 2. Areki----- |
| 562 | Do you take coffee? | 1.Yes | 2.No |
| 563 | If yes for Q559, how Often do you drink coffee? | --------/day | ----/week |
| 564 | If yes for Q559, how many cups? | --------/day | ----/week |
| 565 | On average how many times per day did you take food in the last three months? |  | --------- |
| 566 | Which of the following meal never skip after pregnancy | 1.Breakfast | 2.Lunch 3.Dinner |

Table 20: Environmental factors

Table 21: Household Food Insecurity Access Scale (HFIAS) Measurement Tool

| **S.No** | **Variable** | **Response** | **Skip** |
| --- | --- | --- | --- |
| **801** | In the past four weeks, did you worry that your household would not have enough food? | 1. Yes 2. No | If no for Q801 go to Q802 |
|  | If yes for Q801, how often did this happen? | 1 = Rarely (once or twice in the past four weeks)  2 = Sometimes (3-10 times in the past four weeks)  3 = Often (>10times in the past four weeks) |  |
| **802** | In the past four weeks, were you or any household member not able to eat the kinds of foods you preferred because of a lack of resources? | 1. Yes 2. No | If no for Q802 go to Q803 |
|  | If yes for Q802, how often did this happen? | 1 = Rarely (once or twice in the past four weeks)  2 = Sometimes (3-10 times in the past four weeks)  3 = Often (>10times in the past four weeks) |  |
| **803** | In the past four weeks, did you or any household member have to eat a limited variety of foods due to a lack of resources? | 1. Yes 2. No | If no for Q803 go to Q804 |
|  | If yes for Q803, how often did this happen? | 1 = Rarely (once or twice in the past four weeks)  2 = Sometimes (3-10 times in the past four weeks)  3 = Often (>10times in the past four weeks) |  |
| **804** | In the past four weeks, did you or any household member have to eat some foods that you really did not want to eat because of lack of resource to obtain other types of food? | 1. Yes 2. No | If no for Q804 go to Q805 |
|  | If yes for Q804, how often did this happen? | 1 = Rarely (once or twice in the past four weeks)  2 = Sometimes (3-10 times in the past four weeks)  3 = Often (>10times in the past four weeks) |  |
| **805** | In the past four weeks, did you or any household member have to eat a smaller meal than you felt you needed because there was not enough food? | 1. Yes 2. No | If no for Q805 go to Q806 |
|  | If yes for Q805, how often did this happen? | 1 = Rarely (once or twice in the past four weeks)  2 = Sometimes (3-10 times in the past four weeks)  3 = Often (>10times in the past four weeks) |  |
| **806** | In the past four weeks, did you or any other household member have to eat fewer meals in a day because there was not enough food? | 1. Yes 2. No | If no for Q806 go to Q807 |
|  | If yes for Q806, how often did this happen? | 1 = Rarely (once or twice in the past four weeks)  2 = Sometimes (3-10 times in the past four weeks)  3 = Often (>10times in the past four weeks) |  |
| **807** | In the past four weeks, was there ever no food to eat of any kind in your household because of lack of resources to get food? | 1. Yes 2. No | If no for Q807 go to Q808 |
|  | If yes for Q807, how often did this happen? | 1 = Rarely (once or twice in the past four weeks)  2 = Sometimes (3-10 times in the past four weeks)  3 = Often (>10times in the past four weeks) |  |
| **808** | In the past four weeks, did you or any household member go to sleep at night hungry because there was not enough food? | 1. Yes 2. No | If no for Q808 go to Q809 |
|  | If yes for Q808, how often did this happen? | 1 = Rarely (once or twice in the past four weeks)  2 = Sometimes (3-10 times in the past four weeks)  3 = Often (>10times in the past four weeks) |  |
| **809** | In the past four weeks, did you or any household member go a whole day and night without eating anything because there was not enough food? | 1. Yes 2. No |  |
|  | If yes for Q809, how often did this happen? | 1 = Rarely (once or twice in the past four weeks)  2 = Sometimes (3-10 times in the past four weeks)  3 = Often (>10times in the past four weeks) |  |

Table 23: Maternal Knowledge on diet during pregnancy

| **901** | The amount of food intake should be increased during pregnancy? | 1.Yes | 2.No |
| --- | --- | --- | --- |
| **902** | Frequency of food intake should be increased during pregnancy? | 1.Yes | 2.No |
| **903** | How many times a pregnant woman should take meal per day? |  |  |
| **904** | Pregnant women should take variety of foods? | 1.Yes | 2.No |
| **905** | Which foodstuffs pregnant woman should take every day? List it |  |  |
| **906** | What are the benefits of taking balanced diet to the mother? | ----- |  |
| **907** | What are the benefits of taking balanced diet to the fetus? | ----- |  |
| **908** | Pregnant woman should take iron/folic acid supplement? | 1.Yes | 2.No |
| **909** | How many iron/folic acid supplements should be taken daily? | ------- |  |
| **910** | How long iron/folic acid supplement should be taken? | ----- |  |
| **911** | What are the benefits of taking iron/folic acid supplement during pregnancy? | ---- |  |
| **912** | Which salt is important during pregnancy? | ------ |  |
| **913** | What are the benefits of taking iodized salt? | ------ |  |
| **914** | Pregnant women should take rest? | 1.Yes | 2.No |
| **915** | Pregnant women should reduce work load? | 1.Yes | 2.No |

Table 24: Attitude questions

| **S.No** | Variables | Response | | | | |
| --- | --- | --- | --- | --- | --- | --- |
| **1001** | Pregnant woman should eat variety of foods from different food groups. | 1.Strongly agree | 2.Agree | 3.Neutral | 4.Disagree | 5.Strongly disagree |
| **1002** | Pregnant woman should eat first at mealtimes. | 1.Strongly agree | 2.Agree | 3.Neutral | 4.Disagree | 5.Strongly disagree |
| **1003** | A pregnant woman should eat at least one additional meal. | 1.Strongly agree | 2.Agree | 3.Neutral | 4.Disagree | 5.Strongly disagree |
| **1004** | It is acceptable for pregnant women to skip meals if they do not feel hungry. | 1.Strongly agree | 2.Agree | 3.Neutral | 4.Disagree | 5.Strongly disagree |
| **1005** | It is possible for a pregnant woman to eat any type of food she wants. | 1.Strongly agree | 2.Agree | 3.Neutral | 4.Disagree | 5.Strongly disagree |
| **1006** | Pregnant woman should consume fruits and vegetables five times a daily | 1.Strongly agree | 2.Agree | 3.Neutral | 4.Disagree | 5.Strongly disagree |
| **1007** | Pregnant woman should eat animal products and legumes three times a day. | 1.Strongly agree | 2.Agree | 3.Neutral | 4.Disagree | 5.Strongly disagree |
| **1008** | Pregnant woman should eat potatoes, injera, bread, etc. five times per day. | 1.Strongly agree | 2.Agree | 3.Neutral | 4.Disagree | 5.Strongly disagree |
| **1009** | Pregnant woman should eat foods containing cooking oil and butter on a frequent basis. | 1.Strongly agree | 2.Agree | 3.Neutral | 4.Disagree | 5.Strongly disagree |
| **1010** | Pregnant woman who rests and sleeps during the daytime is lazy and unproductive. | 1.Strongly agree | 2.Agree | 3.Neutral | 4.Disagree | 5.Strongly disagree |
| **1011** | Pregnant woman should have 1-2 hours of rest every day | 1.Strongly agree | 2.Agree | 3.Neutral | 4.Disagree | 5.Strongly disagree |
| **1012** | Pregnant woman should have at least 8 hours of sleep per night | 1.Strongly agree | 2.Agree | 3.Neutral | 4.Disagree | 5.Strongly disagree |
| **1013** | Pregnant woman can perform hard work throughout the day | 1.Strongly agree | 2.Agree | 3.Neutral | 4.Disagree | 5.Strongly disagree |
| **1014** | Pregnant women should use mosquito net during sleep | 1.Strongly agree | 2.Agree | 3.Neutral | 4.Disagree | 5.Strongly disagree |
| **1015** | Pregnant women should not gain weight, since it can cause problems during the delivery process. | 1.Strongly agree | 2.Agree | 3.Neutral | 4.Disagree | 5.Strongly disagree |
| **1016** | Pregnant women should take iron/folic acid supplements. | 1.Strongly agree | 2.Agree | 3.Neutral | 4.Disagree | 5.Strongly disagree |
| **1017** | Pregnant women should use iodized salt. | 1.Strongly agree | 2.Agree | 3.Neutral | 4.Disagree | 5.Strongly disagree |
| **1018** | Pregnant women should drink plenty of water every day. | 1.Strongly agree | 2.Agree | 3.Neutral | 4.Disagree | 5.Strongly disagree |
| **1019** | Eating a healthy diet during pregnancy is beneficial for woman’s health | 1.Strongly agree | 2.Agree | 3.Neutral | 4.Disagree | 5.Strongly disagree |
| **1020** | Eating a healthy diet during pregnancy is beneficial for the fetus’s health | 1.Strongly agree | 2.Agree | 3.Neutral | 4.Disagree | 5.Strongly disagree |

Table 25: Belief about health belief model constructs

| **S.No** | **Variable** | **Response** | |
| --- | --- | --- | --- |
| **1101** | Women perceived that they are threatened to malnourishment when they had inadequate dietary intake | 1.Yes | 2.No |
| **1102** | Women perceived that they are threatened to have IUGR when they had inadequate dietary intake | 1.Yes | 2.No |
| **1103** | Women perceived that they are threatened to have LBW, when they had inadequate dietary intake | 1.Yes | 2.No |
| **1104** | Women perceived that they are threatened to excessive bleeding during delivery when they had inadequate dietary intake | 1.Yes | 2.No |
| **1105** | Women perceived that they are threatened to have preterm birth when they had inadequate dietary intake | 1.Yes | 2.No |
| **1106** | Women perceived that they are threatened to have health problem, when they had inadequate dietary intake | 1.Yes | 2.No |
| **1107** | Women perceived that malnourishment is sever | 1.Yes | 2.No |
| **1108** | Women perceived that the consequences of IUGR are sever | 1.Yes | 2.No |
| **1109** | Women perceived that the consequences of LBW are sever | 1.Yes | 2.No |
| **1110** | Women know that the consequences of preterm birth are sever | 1.Yes | 2.No |
| **1111** | Women know that excessive bleeding is life threatening | 1.Yes | 2.No |
| **1112** | Women know the benefit of adequate dietary intake to improve nutritional status | 1.Yes | 2.No |
| **1113** | Women know the benefit of adequate dietary intake to improve fetal growth | 1.Yes | 2.No |
| **1114** | Women know the benefit of adequate dietary intake to improve birth weight | 1.Yes | 2.No |
| **1115** | Women know the benefit of adequate dietary intake to prevent preterm birth | 1.Yes | 2.No |
| **1116** | Women know the benefit of adequate dietary intake to prevent excessive bleeding | 1.Yes | 2.No |
| **1117** | Women perceived that preparing the required foods during pregnancy takes a lot of time**.** | 1.Yes | 2.No |
| **1118** | My husband is recommending that I pay attention to my nutrition during pregnancy. | 1.Yes | 2.No |
| **1119** | Other family members are recommending that I pay attention to my nutrition during pregnancy. | 1.Yes | 2.No |
| **1120** | Women perceived that she cannot prepare balanced diet | 1.Yes | 2.No |
| **1121** | Women perceived that required foodstuffs aren’t available | 1.Yes | 2.No |

Table 26: Belief about theory of planned behavior constructs

| **S.No** | **Variable** | | **Response** | | | | |
| --- | --- | --- | --- | --- | --- | --- | --- |
|  | **Behavioral intention** | | | | | | |
| **1201** | You intend to eat healthy balanced diet daily during pregnancy | 1.Strongly agree | | 2.Agree | 3.Neutral | 4.Disagree | 5.Strongly disagree |
| **1202** | You intend to take additional meals daily during pregnancy | 1.Strongly agree | | 2.Agree | 3.Neutral | 4.Disagree | 5.Strongly disagree |
| **1203** | You intend to take iron/folate supplement daily during pregnancy | 1.Strongly agree | | 2.Agree | 3.Neutral | 4.Disagree | 5.Strongly disagree |
| **1204** | You intend to use iodized salt during pregnancy | 1.Strongly agree | | 2.Agree | 3.Neutral | 4.Disagree | 5.Strongly disagree |
| **1205** | You intend to reduce workload during pregnancy | 1.Strongly agree | | 2.Agree | 3.Neutral | 4.Disagree | 5.Strongly disagree |
| **1206** | You intend to use mosquito net daily during pregnancy | 1.Strongly agree | | 2.Agree | 3.Neutral | 4.Disagree | 5.Strongly disagree |
|  | **Perceived behavioral control** | | | | | | |
| **1207** | You should confident that you could eat a healthy balanced diet during pregnancy | 1.Strongly agree | | 2.Agree | 3.Neutral | 4.Disagree | 5.Strongly disagree |
| **1208** | You should confident that you could take Iron/folate supplement during pregnancy | 1.Strongly agree | | 2.Agree | 3.Neutral | 4.Disagree | 5.Strongly disagree |
| **1209** | You should confident that you could use iodized salt during pregnancy | 1.Strongly agree | | 2.Agree | 3.Neutral | 4.Disagree | 5.Strongly disagree |
|  | **Subjective norms** |  | |  |  |  |  |
| **1210** | Most people who are important to you think that you should eat a healthy balanced diet during pregnancy | 1.Strongly agree | | 2.Agree | 3.Neutral | 4.Disagree | 5.Strongly disagree |
| **1211** | Your husband believes that you should eat healthy balanced diet | 1.Strongly agree | | 2.Agree | 3.Neutral | 4.Disagree | 5.Strongly disagree |
| **1212** | It is expected of you that you eat a healthy balanced diet during pregnancy | 1.Strongly agree | | 2.Agree | 3.Neutral | 4.Disagree | 5.Strongly disagree |

Table 27: Women decision making related questions

| **S. No** | **Questions** | **Response** |
| --- | --- | --- |
| 1301 | Who is in your family usually has final say in decisions in your own health care? | 1. Husband 2. Women alone 3.Both |
| **1302** | Who is in your family usually has final say in decisions in purchasing land? | 1. Husband 2. Women alone 3.Both |
| **1303** | Who is in your family usually has final say in decisions in constructing house? | 1. Husband 2. Women alone 3.Both |
| **1304** | Who is in your family usually has final say in decisions in visiting your family? | 1. Husband 2. Women alone 3.Both |
| **1305** | Who is in your family usually has final say in decisions in purchasing food stuffs? | 1. Husband 2. Women alone 3.Both |
| **1306** | Who pass a final say on your wealth/ earning? | 1. Husband 2. Women alone 3.Both |
| **1307** | Who pass a final say on borrowing money? | 1. Husband 2. Women alone 3.Both |
| **1308** | Who pass a final say on lending animals? | 1. Husband 2. Women alone 3.Both |
| **1309** | Is there any decision which is left for males only? | 1. Husband 2. Women alone 3.Both |
| **1310** | If your answer is yes for Q 1109 mention it |  |

Table 28: Measurements

| S.No | Variable | Response |
| --- | --- | --- |
| 1401 | MUAC | -------cm |
| 1402 | Blood pressure | ---------mmhg |
